# Unique treatment principles in the service of Interdisciplinary rehabilitation treatment – The MDT method

**DOI:** 10.1192/j.eurpsy.2026.11246

**Published:** 2026-07-31

**Authors:** E. Vashdi, R. Popa

**Affiliations:** 1 Yaelcenter, Haifa, Israel; 2 Dinamic Equilibrium, Bucharest, Romania

## Abstract

**Introduction:**

The MDT is a system that implements the integrative therapy perception. The MDT strives for optimal integration of all developmental areas, disciplines, methods, goals, schools, activities etc. So the child is seen as a whole and all developmental areas are considered in unison (not separately).

The integration takes place at different aspects of therapy: evaluation, analysis process, case management, team work, disciplines and methods, therapist, goals and exercises.

**Objectives:**

The implementation of the rehabilitative MDT system goes through a set of unique treatment principles which guide the therapist throughout the practice. Among the principles we can find the platform, managing energy levels, relationship, attention, goal-oriented thinking, emotional safety, treatment structure, rhythm, timing, threshold point, support, dynamic thinking, ecological treatment, proactivity, clarity and commitment.

**Methods:**

The principles are guidelines to be considered together at every point in therapy along side other considerations such as goals, exercises and techniques. It is not an easy task for the therapist since there is no protocol or recipe but collection of guidelines to lead the way.

**Results:**

The principles are divided to basis, major and common. The basis principles are a necessity for the occurrence of the therapy. The major principles have a significant role in the success of the the therapy. The common principles are important as well but are considered smaller in effect size.

**Conclusions:**

Most of the principles are well known within the rehabilitation therapeutic world, the MDT method has created a unique structure and use of those principles as a philosophy of therapy. The lecture will introduce the 21 principles briefly, and demonstrate the use of them via various tools.

**Disclosure of Interest:**

None Declared

